# Supplementary material for: MetaRibo-Seq measures translation in microbiomes
Source: Nat Commun. 2020 Jun 29;11:3268. doi: 10.1038/s41467-020-17081-z (PMC7324362; doi:10.1038/s41467-020-17081-z)
Supplement: Supplementary file 10 — Supplementary Data 7 [file 41467_2020_17081_MOESM10_ESM.zip › File2/Confidence_High_Taxonomy/250014_out.krona.html]

Javascript must be enabled to view this page.

members
magnitude
magnitudeUnassigned
count
unassigned
taxon
rank

250014\_out

2

1
superkingdom
2

1239
phylum
1

1
class
186801

186802
order
1

family
541000
1

1

SRS078176\_contig\_number\_12449
species
1952287


SRS049995\_contig\_number\_contig-100\_1458.81700
1
